# Some Remarks on Rumex Crispus

**Published:** 1890-06

**Authors:** C. Carleton Smith

**Affiliations:** Philada.


### SOME REMARKS ON RUMEX CRISPUS.

C. CARLETON SMITH, M. D., PHILADA.

On page 172 of the *American Homœopathist* for May, Dr. Cardoza gives a very interesting cure which he made with "Rumex crispus." This calls to memory the fact that it is now nearly twenty-five years since I first used this valuable drug. The case was that of a very intelligent lady, principal of a prominent female seminary. When summoned into her presence, I found her lying upon the bed with a handkerchief held tightly to her mouth which she told me she could not remove from its position without a most violent spasmodic cough resulting. The fact was, she could not even breathe the warm air of the room without irritating the air passages to that degree that a fit of coughing was sure to be induced thereby, short, frequent, and sharp. Rumex being the only drug which exactly corresponded with the characteristic symptoms of the case, it was administered in 6th potency in water, in divided doses. The result was a speedily, brilliant, and permanent cure, astonishing the patient and all her friends.

Dr. Cardoza's cure was that of a cough which only obtained during the day, but not at all at night. I was not aware that this peculiar symptom belonged to Rumex, and therefore it ought to be noted down by all homœpathicians for future use. Most of the drugs, be it remembered, that produce cough in any great degree have *night* aggravations of some sort or another, and in greater or lesser degree. But here is a drug that has a dry, teasing cough all day long which disappears when the patient lies down to rest at night. We thank the doctor for calling our attention to it, as it is not found, as far as we know, in the provings of the drug.

The late Dr. Dunham placed Rumex in the following group, viz.: Belladonna, Lachesis, Phosphorus, Causticum, all of these remedies producing symptoms identical in kind. The characteristic of each, he states, being found in the relative *degree* in which each symptom is pronounced in the different remedies, quite as much as in the possession by any one of these of symptoms not produced by the others.

Thus Bell., Lach., and Rumex, produce each a dry cough, induced by tickling in the larynx or trachea, provoked by deep inspiration, by speaking, and by external pressure on the larynx or trachea. Each produces soreness or rawness of the larynx or trachea.

The cough of each is spasmodic and long continued, and is worse at night after retiring. But, apart from the fact that Bell. and Lach. act more upon the larynx, and Rumex more upon the lower part of the trachea, we observe that, in the case of Lachesis, the slightest external pressure on the larynx or trachea produces violent and long-continued spasmodic cough; the patient cannot endure the least constriction in that region, not even the ordinary contact of his clothing. In the case of Bell. not only is cough produced to a moderate extent by pressing upon the larynx, but soreness and pain are experienced, with a sense of internal fullness which at once suggests the presence of acute laryngitis submucosa. In Rumex, on the other hand, there is no sensibility, strictly speaking, of the trachea, but simply such an irritability of the mucous membrane that cough is produced by the change of position induced in that membrane by external pressure upon the trachea. As regards the extent and intensity of this symptom, Rumex holds a lower rank than the other remedies named. But the irritability of the mucous membrane by virtue of which cough is induced by hurried or deep inspiration, or by speaking, while it is common to Bell., Lach., Rumex, and Phosphorus, is produced in the most exalted degree, as we have already seen, by Rumex, which, as regards this symptom, takes first rank. A sensation of rawness or roughness in the larynx, trachea, and bronchi is produced by each of the four remedies above named, but the *locality* and the *degree* in which it is produced vary in such a manner as to serve in some measure as a characteristic of each. It is most marked in Phos. and Bell., less prominent in Rumex, and least of all in Lach. In Bell. and Lach. it is most marked in the larynx; indeed, it is almost confined to that region. Rumex produces it in the trachea and upper part of the bronchi, while Phos. induces it in the whole mucous tract, from the larynx to the smaller bronchi; and, moreover, in the Phos. proving, this "rawness" of the air-passages is accompanied by a no less characteristic sense of weight and constriction of the upper portion of the thorax, which indicates an affection of the finer air-tubes, and of the air vesicles of such a character as seriously to impede the function of respiration. In considering this last symptom we mention Caust. also, which produces "rawness" extending the whole length of the sternum. All five remedies, again, produce *hoarseness;* Phos., Caust., and Bell. most eminently; Rumex less decidedly, and Lach. in a still less degree. Bell. and Lach. apply especially to those complications which involve the fauces and pharynx, and are acute, the one of a sthenic, the other of an asthenic character; Phos. to those of the pulmonary tissues of a definite inflammatory character, and Rumex to certain affections of the lungs and their envelopes, of which the nature is not clearly defined in the proving. They are indicated by pains, generally sub-acute, in the upper part of the lung near the clavicle and axilla, and more frequent in the left than in the right lung. Since administering my first dose, which was in the earlier days of my practice, my experience with the "Yellow Dock" has been quite extensive, which has led me to pronounce it one of the most valuable remedies in our materia medica for affections of the air-passages.

It undoubtedly possesses antipsoric qualities, for it causes a long-lasting eruption of a vesicular nature, which annoys the patient by itching, only when he is disrobing to go to bed. The eruption appears mostly on lower limbs, and stings, itches, and prickles. The aggravation evidently caused, as is the cough of this drug, by exposure to the air, even of the warm room. Mercurius has an aggravation of its eruption in the evening or night, but only comes on when the patient gets cozily fixed in bed and begins to warm up; which is quite an opposite condition.

A strong Sulphur symptom also obtains with this remedy, viz.: Stool is urgent, driving the patient out of bed early in the morning, the passages being brown or black, and quite thin and watery.

This symptom must not be overlooked in cases of children with the Rumex cough, who are suddenly seized with this early morning stool. Sulphur is not the remedy but Rumex is.

Another point, children with the Rumex cough will become extremely cross and peevish, precisely similar to that condition which obtains under Chamomilla. This may lead, by mistake, to the giving of the latter drug, when, in fact, Rumex ought to have been exhibited, which would be followed by a speedy cure. Belonging to this valuable drug, there is a condition which closely corresponds to Lycopodium, viz.: After meals, great flatulency and distention of stomach. Sensation of fullness after eating, with pressure so great that it extends up into the throat.

Another peculiar condition which was evolved in the proving of Rumex, is one that is evidently connected with indigestion, and which does not, to my knowledge, at least, belong so prominently to any other drug. I allude to the "Lump in throat, not relieved by hawking or swallowing; it descends on deglutition, but immediately returns." I have cured many cases of so-called dyspepsia with this remedy in various potencies where the above characteristic was a prominent factor.

Ignatia has a lump in the throat, also, but during the act of deglutition the patient swallows over it, as it is immovable.

The Rumex patient is very restless in his sleep; dreaming of danger and trouble, and wakes up with a morning headache. This reminds us of Bryonia, but with the latter drug the headache does not assert itself on waking up, but as soon as the eyelids are opened.

Aggravations from cold, damp, raw weather, give us a close similarity to Rhus-tox.

